# Universal adversarial attacks on deep neural networks for medical image classification

**DOI:** 10.1186/s12880-020-00530-y

**Published:** 2021-01-07

**Authors:** Hokuto Hirano, Akinori Minagi, Kazuhiro Takemoto

**Affiliations:** Department of Bioscience and Bioinformatics, Kyushu Institute of Technology, Iizuka, Fukuoka 820-8502 Japan

**Keywords:** Deep neural networks, Medical imaging, Adversarial attacks, Security and privacy

## Abstract

**Background:**

Deep neural networks (DNNs) are widely investigated in medical image classification to achieve automated support for clinical diagnosis. It is necessary to evaluate the robustness of medical DNN tasks against adversarial attacks, as high-stake decision-making will be made based on the diagnosis. Several previous studies have considered simple adversarial attacks. However, the vulnerability of DNNs to more realistic and higher risk attacks, such as universal adversarial perturbation (UAP), which is a single perturbation that can induce DNN failure in most classification tasks has not been evaluated yet.

**Methods:**

We focus on three representative DNN-based medical image classification tasks (i.e., skin cancer, referable diabetic retinopathy, and pneumonia classifications) and investigate their vulnerability to the seven model architectures of UAPs.

**Results:**

We demonstrate that DNNs are vulnerable to both nontargeted UAPs, which cause a task failure resulting in an input being assigned an incorrect class, and to targeted UAPs, which cause the DNN to classify an input into a specific class. The almost imperceptible UAPs achieved > 80% success rates for nontargeted and targeted attacks. The vulnerability to UAPs depended very little on the model architecture. Moreover, we discovered that adversarial retraining, which is known to be an effective method for adversarial defenses, increased DNNs’ robustness against UAPs in only very few cases.

**Conclusion:**

Unlike previous assumptions, the results indicate that DNN-based clinical diagnosis is easier to deceive because of adversarial attacks. Adversaries can cause failed diagnoses at lower costs (e.g., without consideration of data distribution); moreover, they can affect the diagnosis. The effects of adversarial defenses may not be limited. Our findings emphasize that more careful consideration is required in developing DNNs for medical imaging and their practical applications.

## Background

Deep neural networks (DNNs) are effective for image classification and are beginning to be applied to medical image diagnosis to empower physicians and accelerate decision making in clinical environments [1]. For example, DNNs have been used to classify skin cancer based on photographic images [2], referable diabetic retinopathy based on optical coherence tomography (OCT) images of the retina [3], and pneumonia based on chest X-ray images [3]. They have demonstrated high diagnostic performances. A meta-analysis [4] has indicated that the diagnostic performance of DNNs is equivalent to that of healthcare professionals.

Despite DNNs’ high performance, their practical application in disease diagnosis is still debatable. High-stake decision making will be based on disease diagnosis. Complex classifiers, including DNNs, can potentially cause catastrophic harm to society because they are often difficult to interpret [5]. More importantly, DNNs present a number of security concerns [6]. Specifically, DNNs are known to be vulnerable to adversarial examples [7, 8], which are input images that cause misclassifications by DNNs and are typically generated by adding specific, imperceptible perturbations to original input images that have been correctly classified using DNNs. The existence of adversarial examples raises questions about DNNs’ generalization ability, reduces model interpretability, and limits deep learning applications in safety- and security-critical environments [9]. In particular, adversarial examples cause not only misdiagnosis but also various social disturbances [10]. The vulnerability of DNNs to adversarial attacks has been claimed in skin cancer [10] and pneumonia classifications based on chest X-ray images [11].

Nevertheless, more focused investigations are needed on DNNs’ vulnerability to adversarial attacks. Previous studies have only considered input-dependent adversarial attacks (i.e., an individual adversarial perturbation is used such that each input image is misclassified). Such adversarial attacks are difficult because they require high computational costs. More realistic adversarial attacks must be further considered. Notably, a single small, image agnostic perturbation, called *universal adversarial perturbation (UAP)*, that can induce DNN failure in most image classification tasks, has been reported [12]. A previous study [12] considered only UAPs for nontargeted attacks, which cause misclassification (i.e., a task failure resulting in an input image being assigned an incorrect class). However, we previously extended the UAPs generating algorithm to enable targeted attacks [13], which caused the DNN to classify an input image into a specific class. UAPs are difficult to detect because such perturbations are extremely small and, hence, do not significantly affect data distributions. UAP-based adversarial attacks can be more straightforward to implement by adversaries in real-world environments. UAPs are vulnerable to security threats in medical image diagnosis; however, UAP vulnerability is still poorly evaluated in DNN-based medical image diagnosis to date. Specifically, many researchers and engineers have simply developed DNNs using transfer learning (by fine-tuning pretrained DNN models with medical images), inspired by famous studies on medical image classification based on DNNs [2, 3] and have applied DNNs to medical image classification without consideration for their vulnerability to UAPs. Additionally, defense strategies against UAPs in DNN-based medical image classification are still poorly investigated, although the vulnerability of DNNs to adversarial attacks indicates the need for strategies to address security concerns (i.e., adversarial defense [8]). Specifically, adversarial retraining is one of the few approaches that could not be defeated thus far [14].

This study aims to evaluate the vulnerability of DNNs to UAPs for medical image classification and to warn against facile applications of DNNs for medical image classification. We focused on representative medical image classifications: skin cancer classification based on photographic images [2], referable diabetic retinopathy classification based on OCT images [3], and pneumonia classification based on chest X-ray images [3]. We obtained DNN models with various architectures for medical image diagnosis inspired by previous studies [2, 3] and investigated their vulnerability to nontargeted and targeted attacks based on UAPs. Moreover, adversarial defense was considered; in particular, we evaluated the increased robustness of DNNs to nontargeted and targeted UAPs using adversarial retraining [12, 14–16], a representative method for adversarial defenses.

## Methods

### Medical image datasets

We used three types of medical images: skin lesion images for skin cancer classification, OCT images for referable diabetic retinopathy classification, and chest X-ray images for pneumonia classification.

In a previous study [2], skin lesion images (red–green–blue color) were obtained from the International Skin Imaging Collaboration (ISIC) 2018 dataset (challenge2018.isic-archive.com/task3/training/), in which the images were classified into seven classes: melanoma (MEL), melanocytic nevus (NV), basal cell carcinoma (BCC), actinic keratosis/Bowens disease (intraepithelial carcinoma; AKIEC), benign keratosis (solar lentigo/seborrheic keratosis/lichen planus-like keratosis; BKL), dermatofibroma (DF), and vascular lesions (VASC). The dataset comprised 10,015 images. We randomly divided these images into 7,000 training images (778 MEL, 4,689 NV, 370 BCC, 229 AKIEC, 764 BKL, 76 DF, and 94 VASC images, respectively) and 3,015 test images (335 MEL, 2016 NV, 144 BCC, 98 AKIEC, 335 BKL, 39 DF, and 48 VASC images, respectively).

The OCT and chest X-ray images (grayscale) were obtained from a previous study [3] (data.mendeley.com/datasets/rscbjbr9sj/3). The OCT images were classified into four classes: choroidal neovascularization with neovascular membrane and associated subretinal fluid (CNV), diabetic macular edema with retinal-thickening-associated intraretinal fluid (DME), multiple drusen present in early age-related macular degeneration (DRUSEN), and normal retina with preserved foveal contour and absence of any retinal fluid/edema (NM). The original dataset comprised 37,455 CNV, 11,598 DME, 8866 DRUSEN, and 51,390 NM images, respectively. We constructed a class-balanced training image set and test image set by randomly selecting 1960 and 840 images per class, without duplicates, respectively. We finally obtained 7840 training and 3360 test images.

The chest X-ray images were classified into binary classes: no pneumonia (NORMAL) or viral or bacterial pneumonia (PNEUMONIA). The original dataset comprised 1583 NORMAL and 4273 PNEUMONIA images. We constructed a class-balanced training image set and test image set by randomly selecting 900 and 270 images per class, without duplicates, respectively. We finally obtained 1800 training and 540 test images.

### Transfer learning methods

Following previous studies [2, 3], we obtained the DNN models using transfer learning; in particular, we fine-tuned DNN models pretrained using the ImageNet dataset [17] with a medical image dataset. We mainly considered the Inception V3 architecture [18], following previous studies. To investigate the effect of model architecture on adversarial robustness, we considered different model architectures: VGG16 [19], VGG19 [19], ResNet50 [20], Inception ResNet V2 [21], DenseNet 121 [22], and DenseNet 169 [22]. For each model architecture, we replaced the original last fully connected (FC) layer with a new FC layer in which the output size is the number of classes. The images were resized to 299 × 299 pixels. All layer parameters were fine-tuned using the training images in a medical image dataset. We used the stochastic gradient descent optimizer with a momentum of 0.9. The batch size and number of epochs were set to 32 and 50, respectively. The learning rates were scheduled based on the number of epochs: 0.001 for ≤ 40 epochs, 1e−4 for 41–45 epochs, and 1e−5 for > 45 epochs. To avoid overfitting, data augmentation was considered: random image rotations with angles ranging between − 5° and 5° and random 5% height and width image shifts. For the skin cancer classification, we adopted oversampling to account for imbalances in the dataset. The transfer learning procedures were performed using Keras (version 2.2.4; Keras.io).

### Universal adversarial perturbations

Simple iterative algorithms [12, 13] were used to generate UAPs for nontargeted and targeted attacks. The algorithms’ details are described in [12, 13]. We used the nontargeted UAP algorithm available in the Adversarial Robustness 360 Toolbox (ART) [23] (version 1.0; github.com/Trusted-AI/adversarial-robustness-toolbox). The targeted UAP algorithm was implemented by modifying the nontargeted UAP algorithm from our previous study in ART [13] (github.com/hkthirano/targeted_UAP_CIFAR10).

The algorithms consider a classifier and generate nontargeted (targeted) UPAs ${\varvec{\rho}}$ from an input image set ${\varvec{X}}$, under the constraint that the $L_{p}$ norm of the perturbation is equal to or less than a small $\xi$ value (i.e., $\user2{ }\left\| {{\varvec{\rho}}_{p} } \right\| \le \xi$). The algorithms start with ${\varvec{\rho}} = {\mathbf{0}}$ (no perturbation) and iteratively update ${\varvec{\rho}}$ by additively obtaining an adversarial perturbation for an input image ${\varvec{x}}$, which is randomly selected from ${\varvec{X}}$ without replacement. These iterative updates continue until the number of iterations reaches a maximum $i_{{\max}}$.

The fast gradient sign method (FGSM) [7] is used to obtain an adversarial perturbation for the input image. Meanwhile, the original UAP algorithm [12] uses the DeepFool method [24]. This is because the FGSM is used for both nontargeted and targeted attacks, and DeepFool requires a higher computational cost than the FGSM and only generates a nontargeted adversarial example for the input image. The FGSM generates the adversarial perturbation for ${\varvec{x}}$ based on the loss gradient [7] with the attack strength parameter $\epsilon$.

Nontargeted and targeted UAPs were generated using the training images in the dataset. The parameter $\epsilon$ was set to 0.0024; cases where $p = 2$ and $\infty$ were considered. The parameter $\xi$ was determined based on the ratio $\zeta$ of the $L_{p}$ norm of the UAP to the average $L_{p}$ norm of an image in the dataset. For the ISIC 2018 (skin lesion image) dataset, the average $L_{\infty }$ and $L_{2}$ norms were 237 and 85,662, respectively. For the OCT image dataset, the average $L_{\infty }$ and $L_{2}$ norms were 253 and 15,077, respectively. For the chest X-ray image dataset, the average $L_{\infty }$ and $L_{2}$ norms were 253 and 40,738, respectively. The parameter $i_{{\max}}$ was set to 15.

To compare the performances of the generated UAPs with those of the random controls, we generated random vectors (random UAPs) sampled uniformly from the sphere of a specified radius [12].

### Vulnerability evaluation

The fooling rate $R_{f}$ and targeted attack success rate $R_{s}$ were computed to evaluate the vulnerability of the DNN models to a nontargeted UAP (${\varvec{\rho}}_{{{\text{nt}}}}$) and targeted UAP (${\varvec{\rho}}_{{\text{t}}}$), respectively. Further, $R_{f}$ for an image set ${\varvec{X}}$ is defined as adversarial images for which predicted labels are inconsistent with the labels predicted from their associated clean images to all images in the set (i.e., the probability that the labels predicted from clean images are inconsistent with the labels predicted from their adversarial images). Let $C\left( {\varvec{x}} \right)\user2{ }$ be an output (class or label) of a classifier (DNN) for an input image $\user2{ x}$, $R_{f} = \left| {\varvec{X}} \right|^{ - 1} \mathop \sum \limits_{{{\varvec{x}} \in {\varvec{X}}}} {\mathbb{I}}\left( {C\left( {\varvec{x}} \right) \ne C\left( {{\varvec{x}} + {\varvec{\rho}}_{{{\text{nt}}}} } \right)} \right)$, where the function ${\mathbb{I}}\left( A \right)$ returns 1 if the condition $A$ is true and 0 otherwise. $R_{s}$ for an image set is the proportion of adversarial images classified into the target class $y$ to all images in the set $R_{s} = \left| {\varvec{X}} \right|^{ - 1} \mathop \sum \limits_{{{\varvec{x}} \in {\varvec{X}}}} {\mathbb{I}}\left( {C\left( {{\varvec{x}} + {\varvec{\rho}}_{{\text{t}}} } \right) = y} \right)$. It is noteworthy that $R_{s}$ has a baseline, defined as $R_{s}$, observed without UAPs. Class (label) composition of image data and prediction performance of DNNs both affect the baseline. In this study, for the OCT and chest X-ray image datasets, the $R_{s}$ baselines of UAPs targeted to a specified class were ~ 25% and ~ 50%, respectively. For the skin lesion dataset, the $R_{s}$ baselines of UAPs targeted to MEL and NV were ~ 10% and ~ 65%, respectively.

Additionally, we obtained the confusion matrices, to evaluate the change in prediction owing to the UAPs for each class. The rows and columns in the matrices represent true and predicted classes, respectively. The confusion matrices were row-normalized to account for an imbalanced dataset (ISIC 2018 dataset); in particular, each cell value was normalized by the number of observations with the same true class (label).

### Adversarial retraining

Adversarial retraining was performed to increase the robustness of the DNN models to UAPs [12, 15]; in particular, the models were fine-tuned with adversarial images. The procedure was described in a previous study [12]. A schematic diagram of the adversarial retraining procedure is shown in Additional file 1: Fig. S1. A brief description is provided here: (1) 10 UAPs against a DNN model were generated with the (clean) training image set; (2) a modified training image set was obtained by randomly selecting half of the training images and combining them with the remaining images in which each image was perturbed by a UAP randomly selected from the 10 UAPs; (3) the model was fine-tuned by performing five additional epochs of training on the modified training image set; (4) a new UAP was generated against the fine-tuned model using the algorithm with the training image set; (5) the UAP $R_{f}$ and $R_{s}$ values for the test images were computed; and steps (1)–(5) were repeated five times.

## Results

### Medical images classification performance

We evaluated the prediction performance of seven DNN models for three medical image datasets. The test and training accuracies of the models for the datasets are summarized in Additional file 1: Table S1. The DNN models achieved good accuracy. For the skin lesion, OCT, and chest X-ray image datasets, the average test accuracies across the seven models were 87.3%, 95.8%, and 98.4%, respectively. Specifically, the test accuracies of Inception V3 models, which were frequently used in previous studies on medical image diagnosis (e.g., [2, 3]), were 87.7%, 95.5%, and 97.6%, respectively. The normalized confusion matrices for the Inception V3 models on the test images are shown in Additional file 1: Fig. S2.

### Nontargeted universal adversarial attacks

**Fig. 1:**
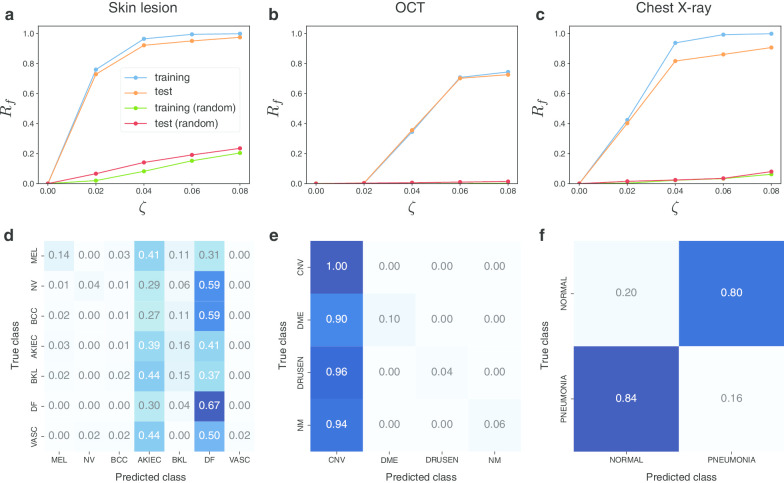
Vulnerability to nontargeted UAPs with $p = 2$. Line plots of the fooling rate $R_{f}$ against Inception V3 model versus perturbation magnitude $\zeta$ for skin lesions (**a**), OCT (**b**), and chest X-ray (**c**) image datasets. Legend label indicates image set used for computing $R_{f}$. Additional argument “(random)” indicates that random UAPs were used instead of UAPs. Normalized confusion matrices for Inception V3 models attacked using UAPs on test images of skin lesions (**d**), OCT (**e**), and chest X-ray (**f**) image datasets are also shown. $\zeta = 4\%$ in **d** and **f**. $\zeta = 6\%$ in **e**

We evaluated the vulnerability of the DNN models to nontargeted UAPs. We first considered Inception V3 models because well-known previous studies on DNN-based medical image classification used the Inception V3 architecture [2, 3]. Figure 1 shows the case of nontargeted UAPs $p = 2$ against the Inception V3 models. The fooling rates $R_{f}$ for both the training and test images increased rapidly with the perturbation magnitude $\zeta$ and reached a high $R_{f}$, despite a low $\zeta$. The UAPs with $\zeta = 4\%$ achieved $R_{f}$ > 80% for the skin lesion (Fig. 1a) and chest X-ray image datasets (Fig. 1c), whereas slightly larger UAPs (with $\zeta = 6\%$) were required to achieve $R_{f}$ ~ 70% for the OCT image dataset (Fig. 1b). Further, $R_{f}$ of the nontargeted UAPs was significantly higher than that of random UAPs. The confusion matrices on test images show that the models classified most images into several specific classes (i.e., dominant classes) due to the UAPs for the skin lesion and OCT image datasets. Specifically, most skin lesion images tended to be classified as AKIEC or DF (Fig. 1d); moreover, most OCT images were classified as CNV (Fig. 1e). For the chest X-ray image dataset, the model incorrectly predicted the true labels (Fig. 1f). A high $R_{f}$ at low $\zeta$ and dominant labels was observed in the case of UAP with $p = \infty$ against the Inception V3 models for all medical image datasets (Additional file 1: Fig. S3). However, the skin lesion images tended to be classified into broader classes: BCC, AKIEC, BKL, or DF (Additional file 1: Fig. S3D).

We also considered other models to evaluate whether the vulnerability to nontargeted UAPs depends on model architectures. Table 1 shows $R_{f}$ of the UAPs against the DNN models for the test images in the medical image datasets. Overall, a vulnerability to nontargeted UAPs was observed independent of model architectures; in particular, the small UAPs ($\zeta = 4\%$ for the skin lesions and chest X-ray image datasets, and $\zeta = 6\%$ for the OCT image dataset) achieved a high $R_{f}$ (70–90%). The UAPs’ $R_{f}$ were significantly higher than those of the random UAPs. However, $R_{f}$ partially depends on model architectures; specifically, $R_{f}$ of the UAPs against the VGG16 and VGG19 models were ~ 50% for the chest X-ray image dataset, whereas those of the UAPs against the other models were between 70 and 80%. This indicates that the models classified images into either NORMAL or PNEUMONIA. In the case of UAPs with $p = 2$, the VGG16 and VGG19 models classified most test images into PNEUMONIA and NORMAL, respectively (Additional file 1: Fig. S4). In the case of UAPs with $p = \infty$, both the VGG16 and VGG19 models predicted most of the test images as NORMAL. This indicates that the confusion matrix patterns (dominant classes) might change according to the model architecture and $p$. Additionally, a change in confusion matrix patterns (on test images) was observed in the skin lesions and OCT image datasets. For example, the VGG16 model classified most skin lesion images into BKL owing to the UAP with $\zeta = 4\%$ and $p = 2$ (Additional file 1: Figure S5A), whereas the Inception V3 models classified them into AKIEC or DF (Fig. 1d). The ResNet 50 model classified most OCT images into DME owing to the UAP with $\zeta = 6\%$ and $p = 2$ (Additional file 1: Fig. S5B), whereas Inception V3 models classified them into CNV (Fig. 1e).

**Table 1:** Fooling rates $R_{f}$ (%) of nontargeted UAPs against various DNN models for test images of skin lesions, OCT, and chest X-ray image datasets

Model architecture	Skin lesions		OCT		Chest X-ray	
	$p = 2$	$p = \infty$	$p = 2$	$p = \infty$	$p = 2$	$p = \infty$
Inception V3	92.2 (14.1)	90.0 (11.8)	70.2 (1.0)	73.9 (3.4)	81.7 (2.4)	79.8 (3.0)
VGG16	87.6 (4.9)	86.4 (3.5)	72.4 (0.2)	74.9 (1.8)	49.8 (2.2)	50.0 (2.2)
VGG19	89.2 (5.2)	87.0 (3.7)	72.8 (0.4)	74.7 (2.1)	49.3 (3.9)	49.3 (4.4)
ResNet50	91.9 (11.6)	87.9 (10.1)	71.2 (1.1)	74.8 (5.4)	72.6 (7.2)	73.0 (7.4)
Inception ResNet V2	94.5 (16.7)	90.3 (15.2)	69.6 (1.4)	74.0 (3.2)	78.0 (2.6)	77.0 (3.3)
DenseNet 121	93.8 (12.0)	82.9 (10.2)	68.8 (1.3)	73.0 (3.6)	69.8 (3.9)	71.7 (4.1)
DenseNet 169	93.8 (11.7)	84.2 (9.1)	50.3 (1.3)	72.3 (4.0)	67.6 (2.8)	71.3 (3.7)

$\zeta = 4\%$ for the skin lesions and chest X-ray image datasets. $\zeta = 6\%$ for the OCT image dataset. Values in brackets are $R_{f}$ of random UAPs (random controls)

**Fig. 2:**
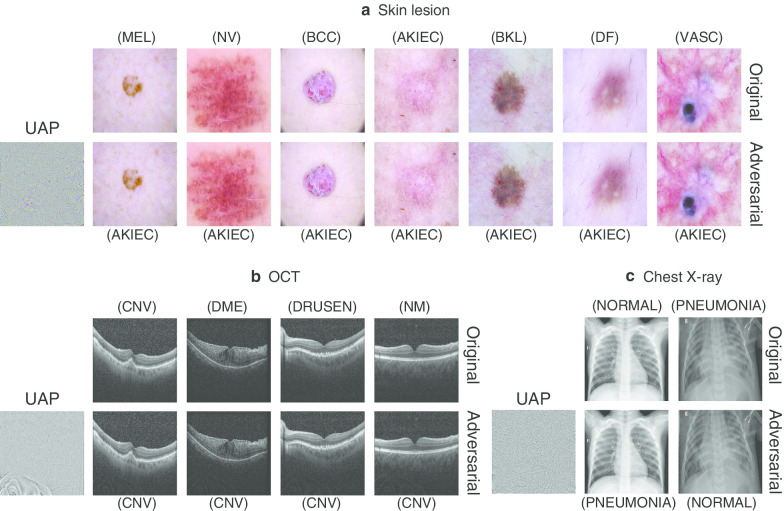
Nontargeted UAPs with $p = 2$ against Inception V3 models and their adversarial images for skin lesions (**a**), OCT (**b**), and chest X-ray image datasets (**c**). Further, $\zeta = 4\%$ in **a** and **c**. $\zeta = 6\%$ in **b**. Labels in brackets beside the images are the predicted classes. The original (clean) images are correctly classified into their actual labels. UAPs are emphatically displayed for clarity; in particular, each UAP is scaled by a maximum of 1 and minimum of 0

We investigated whether the nontargeted UAPs were perceptible. As a representative example, the nontargeted UAPs with $p = 2$ against the Inception V3 models and examples of adversarial images for the medical image datasets are shown in Fig. 2. The UAPs with $\zeta = 4\%$ for the skin lesions and chest X-ray image datasets and with $\zeta = 6\%$ for the OCT image dataset were almost imperceptible. The models predicted the original images as their actual classes; however, they classified the adversarial images into incorrect classes owing to the nontargeted UAPs. The UAPs with $p = \infty$ and those against the other DNN models were also almost imperceptible for the skin lesion (Additional file 1: Fig. S6), OCT (Additional file 1: Fig. S7), and chest X-ray image datasets (Additional file 1: Fig. S8).

Moreover, we found that different UAP patterns were observed in the different model architectures for each medical image dataset (Additional file 1: Figs. S6–S8). We hypothesized that the UAPs have no transferability, which indicates that UAPs generated based on DNNs with one model architecture can be used to deceive DNNs with another model architecture and to evaluate the transferability of UAPs. As expected, transferability was not confirmed for the OCT (Additional file 1: Table S3) and chest X-ray image datasets (Additional file 1: Table S4); however, a weak transferability of UAPs was observed in the skin lesions image dataset (Additional file 1: Table S5). Specifically, the nontargeted UAPs with $p = 2$ generated based on the Inception V3 models achieved $R_{f}$ of approximately 45%, ~ 2%, and ~ 10% on average against the DNNs with another model architecture for the skin lesions, OCT, and chest X-ray image datasets, respectively.

### Targeted universal adversarial attacks

**Fig. 3:**
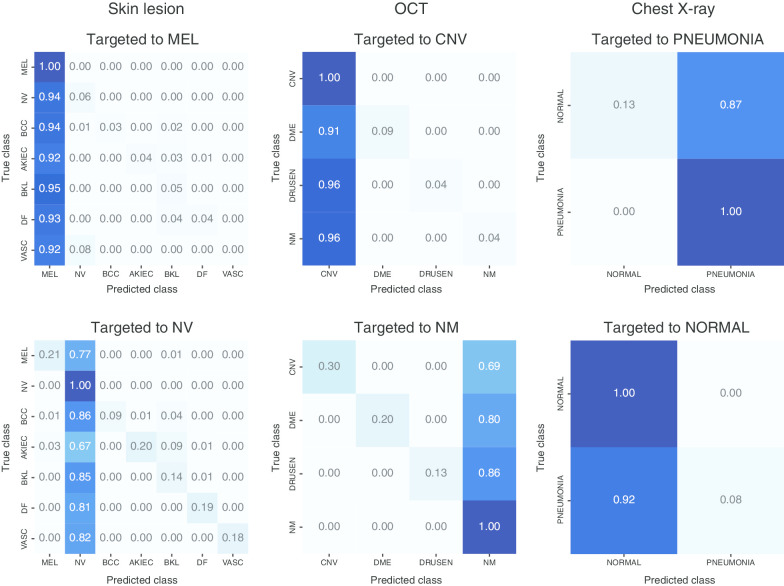
Normalized confusion matrices for Inception V3 models attacked with targeted UPAs with $p = 2$ on test images in skin lesions (left panels), OCT (middle panels), and chest X-ray image datasets (right panels). Further, $\zeta = 2\%$ for skin lesions and chest X-ray image datasets, and $\zeta = 6\%$ for OCT image dataset

We have developed targeted UAPs in our previous study [13]. We evaluated whether the DNNs are vulnerable not only to nontargeted UAPs but also to targeted UAPs (i.e., whether UAPs can control DNN outputs). Table 2 shows the targeted attack success rates $R_{s}$ of the UAPs with $p = 2$ against the DNN models for the test images in the medical image datasets. As representative examples, we considered targeted attacks to be the most significant case and the control in each medical image dataset. For skin lesion image datasets, targeted attacks on MEL and NV were considered. For the OCT image dataset, targeted attacks on CNV and NM were considered. For the chest X-ray image dataset, targeted attacks on PNEUMONIA and NORMAL were considered. Overall, a high (> 85%) $R_{s}$ was observed regardless of the model architecture, despite small UAPs (with $\zeta = 2\%$ for the skin lesions and chest X-ray image datasets, and $\zeta = 6\%$ for the OCT image dataset). Furthermore, the confusion matrices (Fig. 3) indicate that the UAP-based targeted attacks were successful: most ($R_{s} \%$ of) test images were classified into the targeted class owing to the UAPs (Table 2). However, a smaller $R_{s}$ was partially observed according to the model architectures and datasets. For the skin lesions image dataset, $R_{s}$ of the UAPs against VGG16 (~ 40%) and VGG19 (~ 65%) models were lower than those (~ 90%) of the UAPs against the other models. For the targeted attacks on NM in the OCT image dataset, $R_{s}$ (30–40%) of the UAPs against the VGG and DensNet models were lower than those (~ 85%) of the UAPs against the other models. Further, $R_{s}$ of random UAPs was almost equivalent to those of the baselines. The $R_{s}$ values of the UAPs were significantly higher than those of the random UAPs. Furthermore, a high $R_{s}$ for a small $\zeta$ was observed for the targeted UAPs with $p = \infty$ (Additional file 1: Table S2). However, $R_{s}$ for targeted attacks on MEL was lower overall than $R_{s}$ of the UAPs with $p = 2$. For example, $R_{s}$ of the UAPs with $p = 2$ and $p = \infty$ against the Inception V3 model were ~ 95% and ~ 75%, respectively.

**Table 2:** Targeted attack success rates $R_{s}$ (%) of targeted UAPs with $p = 2$ against various DNN models to each target class

Model architecture/target class	Skin lesions		OCT		Chest X-ray	
	NV	MEL	NM	CNV	NORMAL	PNEUMONIA
Inception V3	93.3 (65.6)	94.4 (12.2)	84.1 (25.7)	95.9 (24.8)	96.1 (52.8)	93.3 (47.2)
VGG16	89.6 (71.7)	40.4 (8.3)	32.4 (25.4)	97.7 (24.9)	95.6 (50.2)	95.0 (49.8)
VGG19	91.6 (72.1)	64.6 (8.7)	41.2 (25.9)	97.5 (24.9)	97.6 (51.7)	95.2 (48.3)
ResNet50	97.9 (66.5)	92.4 (11.8)	84.9 (25.8)	98.5 (24.5)	95.7 (53.5)	95.2 (46.5)
Inception ResNet V2	92.4 (61.0)	97.3 (16.1)	84.5 (25.6)	96.2 (24.7)	98.3 (53.1)	93.9 (46.9)
DenseNet 121	92.1 (65.2)	90.5 (13.4)	41.8 (25.3)	88.1 (24.7)	94.8 (51.9)	92.0 (48.1)
DenseNet 169	92.9 (65.8)	92.9 (12.2)	41.7 (25.0)	92.7 (24.2)	95.7 (52.0)	93.1 (48.0)

$R_{s}$ was for test images, $\zeta = 2\%$ for the skin lesions and chest X-ray image datasets, and $\zeta = 6\%$ for the OCT image dataset. Values in brackets are $R_{s}$ of random UAPs (random controls)

**Fig. 4:**
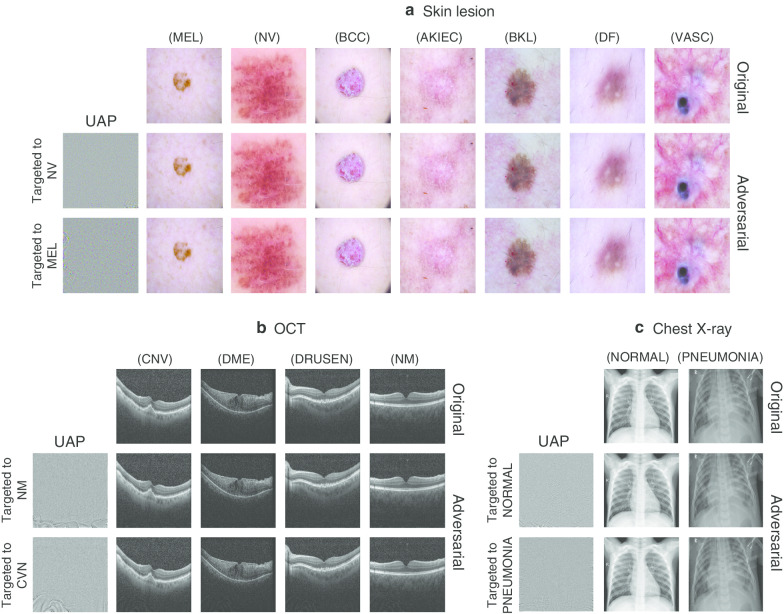
Targeted UAPs with $p = 2$ against Inception V3 models and their adversarial images for skin lesions (**a**), OCT (**b**), and chest X-ray image datasets. Further, $\zeta = 2\%$ in **a** and **c**. $\zeta = 6\%$ in **b**. Labels in brackets beside the images are predicted classes. Original (clean) images were correctly classified into their actual labels. Adversarial images were classified into the target classes. UAPs are emphatically displayed for clarity; in particular, each UAP is scaled by a maximum of 1 and minimum of 0

We investigated whether the targeted UAPs were perceptible. As a representative example, the targeted UAPs with $p = 2$ against the Inception V3 models and examples of adversarial images for the medical image datasets are shown in Fig. 4. The targeted UAPs with $\zeta = 2\%$ for the skin lesions and chest X-ray image datasets and $\zeta = 6\%$ for the OCT image dataset were also almost imperceptible. The models predicted the original images as their actual classes; however, they classified the adversarial images into the targeted class owing to the UAPs. The UAPs with $p = \infty$ and those against the other DNN models were also almost imperceptible. For the skin lesion image dataset, Additional file 1: Figures S9 and S10 show the targeted attacks on NV and MEL, respectively. For the OCT image dataset, Additional file 1: Figures S11 and S12 show the targeted attacks on NM and CNV, respectively. For the chest X-ray image dataset, Additional file 1: Figures S13 and S14 show the targeted attacks on NORMAL and PNEUMONIA, respectively.

We also evaluated whether UAP patterns depend on model architectures and found that they did so for each medical image dataset (Additional file 1: Figs. S9–S14). The non-transferability of UAPs was also confirmed for the skin lesions (Additional file 1: Table S6), OCT (Additional file 1: Table S7), and chest X-ray image datasets (Additional file 1: Table S8); specifically, $R_{s}$ observed when the targeted UAPs with $p = 2$ generated based on the Inception V3 model that attacked the DNN models with another architecture were almost equivalent to their baselines of $R_{s}$ ~ 10%, ~ 25%, and ~ 50% for the skin lesions, OCT, and chest X-ray image datasets, respectively.

### Adversarial retraining

We analyzed the usefulness of adversarial retraining against universal adversarial attacks (both nontargeted and targeted UAPs). We considered Inception V3 models because well-known previous studies on DNN-based medical image classification used the Inception V3 architecture [2, 3].

**Fig. 5:**
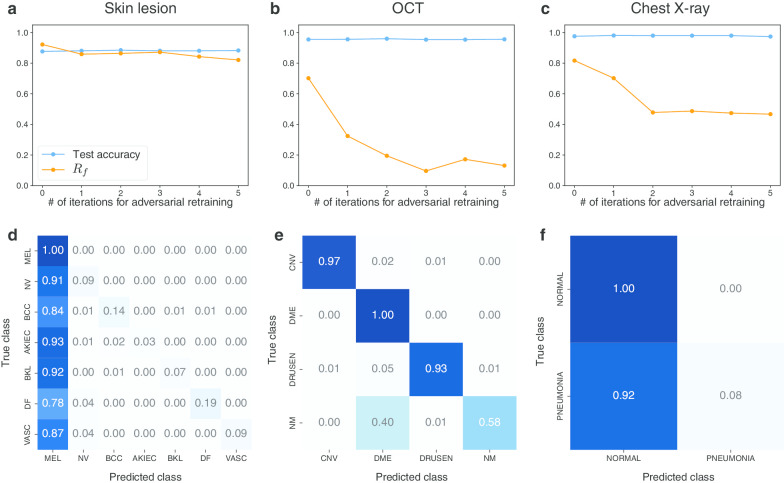
Effect of adversarial retraining on robustness of nontargeted UAPs with $p = 2$ against Inception V3 models for skin lesions, OCT, and chest X-ray image datasets. $\zeta = 4\%$ for the skin lesions and chest X-ray image datasets. $\zeta = 6\%$ for OCT image dataset. The top panels indicate the scatter plots of fooling rate $R_{f}$ (%) of UAPs versus number of iterations for adversarial retraining. Bottom panels indicate normalized confusion matrices for fine-tuned models obtained after five iterations of adversarial retraining. These confusion matrices are on adversarial test images

Figure 5 shows the effect of adversarial retraining on $R_{f}$ of nontargeted UAPs with $p = 2$ against Inception V3 models for the skin lesions, OCT, and chest X-ray image datasets, $\zeta = 4\%$ for the skin lesions and chest X-ray image datasets, and $\zeta = 6\%$ for the OCT image dataset. Adversarial retraining did not affect test accuracy. For the OCT image dataset, $R_{f}$ decreased with the adversarial retraining iterations; specifically, $R_{f}$ decreased from 70.2 to 13.1% after five iterations (Fig. 5b); however, ~ 40% of the NM images were still classified into an incorrect class (DME, Fig. 5e). The adversarial retraining effect on $R_{f}$ was limited for the skin lesions (Fig. 5a) and chest X-ray image datasets (Fig. 5b). For the chest X-ray image dataset, $R_{f}$ decreased from 81.7 to 46.7%. A $R_{f}$ of ~ 50% indicates that the model classified most images into either one of two classes; specifically, most images were classified into NORMAL at the fifth iteration (Fig. 5f). For the skin lesions image dataset, no remarkable decrease in $R_{f}$ due to adversarial retraining was confirmed; specifically, $R_{f}$ decreased from 92.2 to 82.1% (Fig. 5a). Most images were classified into MEL at the fifth iteration (Fig. 5c). However, the dominant classes changed for each iteration. For example, the dominant classes were AKIEC and BKL at the third and fourth iterations, respectively (Fig. S15 in Additional file 1).

**Fig. 6:**
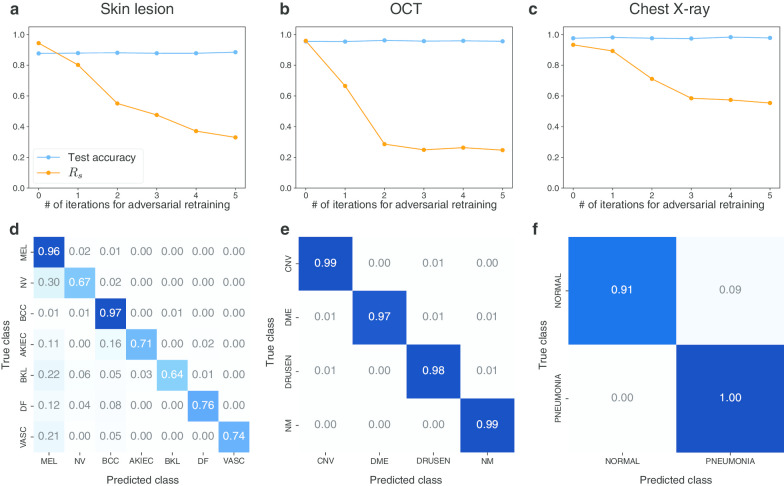
Effect of adversarial retraining on robustness of targeted UAPs with $p = 2$ against Inception V3 models for skin lesions, OCT, and chest X-ray image dataset. $\zeta = 2\%$ for skin lesion and chest X-ray image datasets. $\zeta = 6\%$ for OCT image dataset. Top panels indicate scatter plots of targeted attack success rate $R_{s}$ (%) of UAPs versus number of iterations for adversarial retraining. Bottom panels indicate normalized confusion matrices for fine-tuned models obtained after five iterations of adversarial retraining. These confusion matrices are on adversarial test images

Figure 6 shows the effect of adversarial retraining on the $R_{s}$ of targeted UAPs with $p = 2$ against the Inception V3 models for the skin lesions, OCT, and chest X-ray image datasets. As representative examples, we considered targeted attacks on the most significant cases, namely, MEL, CNV, and PNEUMONIA for the skin lesions, OCT, and chest X-ray image datasets, respectively; $\zeta = 2\%$ for the skin lesions and chest X-ray image datasets; and $\zeta = 6\%$ for the OCT image dataset. Adversarial retraining did not affect the test accuracy and reduced $R_{s}$ for all medical image datasets (Fig. 6a–c). For the OCT and chest X-ray image dataset, $R_{s}$ decreased from ~ 95% to the baseline $R_{s}$ (~ 25% and ~ 50%, respectively) after five iterations. For the skin lesions image dataset, $R_{s}$ decreased from ~ 95 to ~ 30%; however, $R_{s}$ at the fifth iteration was higher than the baseline (~ 10%). The confusion matrices (Fig. 6d–f) indicated that adversarial retraining was useful against UAP-based targeted attacks: most images were correctly classified into the original classes despite the adversarial attacks. However, the effect of adversarial retraining was partially limited for the skin lesions image dataset. For example, 30% of the NV images were still classified into the target class (MEL) despite five iterations of adversarial retraining (Fig. 6c). Furthermore, ~ 20% of the BKL and VASC images were still classified into the target class.

## Discussion

We showed the vulnerability of the DNN models for medical image classification to small UAPs. Previous studies [10, 11] have indicated the vulnerability to adversarial attacks toward medical DNNs; however, they were limited to input image-dependent perturbations. In this study, we demonstrated that almost imperceptible UAPs caused DNN misclassifications. Unlike previous assumptions, these results indicate that a DNN-based medical image diagnosis is easier to deceive. Adversaries can result in failed DNN-based medical image diagnoses at lower costs (i.e., using a single perturbation). Specifically, they do not need to consider the distribution and diversity of input images when attacking DNNs using UAPs, as UPAs are image agnostic.

We demonstrated that nontargeted attacks based on UAPs were possible (Figs. 1 and 2, Table 1). Most images were classified into a few specific classes for the skin lesions and OCT image (multiclass) datasets. This result is consistent with the existence of dominant classes in UAP-based nontargeted attacks [12]. For the skin lesions image dataset, the AKIEC and DF dominant classes observed in this study may be owing to the imbalanced dataset. The number of AKIEC and DF images is relatively lower than that of the other class images. As the algorithm considers maximizing $R_{f}$, a relatively large $R_{f}$ is achieved when all inputs are classified into AKIEC and DF owing to UAPs. The use of imbalanced datasets may be one of the causes of vulnerability to UAPs. To avoid this problem, domain adaptation [25, 26] may be useful. For the OCT image (binary-class) dataset, the DNN models wrongly predicted the actual labels because of $R_{f}$ maximization; however, the existence of dominant classes was partially confirmed according to the model architecture. These misclassifications result in false positives and false negatives in medical diagnosis. False positives may cause unwanted mental stress to patients, whereas false negatives may result in significant misdiagnoses involving human lives; specifically, they fail to perform early detection and render therapeutic strategies difficult. Moreover, they can cause the social credibility of medical doctors and medical organizations to be undermined.

The transferability of nontargeted UAPs across model architectures was limited (Additional file 1: Tables S3–S5). This indicates that UAPs are architecture-specific, which is inconsistent with a previous study [12]. This discrepancy might be due to differences in the image datasets. Specifically, the number of classes (2–7) in the medical image datasets was lower than that (1000) of the dataset used in the previous study. This study partly considered grayscale images, whereas the previous study used colored images only. Transferability may be observed in datasets comprising colored images with more classes. In fact, a weak transferability was observed for the skin lesions image dataset (Additional file 1: Table S5).

Furthermore, we showed that targeted attacks based on UAPs were possible in medical image diagnosis (Figs. 3 and 4, Table 2), although the UAPs were not transferable across model architectures (Additional file 1: Tables S6–S8). The results imply that adversaries can control DNN-based medical image diagnoses. As targeted attacks are more realistic, they may result in more significant security concerns compared with nontargeted attacks. In particular, adversaries can obtain any diagnosis; specifically, they can intentionally cause not only problems resulting from misdiagnosis, but also various social disturbances. As mentioned in a previous study [10], adversarial attacks can be used for insurance fraud, as well as drug and device approval adjustments, thereby fraudulently providing and obtaining high-quality care when DNNs are used for decision making.

We considered adversarial retraining, which is known to be an effective method for adversarial defenses [14], to reduce the vulnerability to UAPs. However, the effect of adversarial retraining was limited for nontargeted UAPs (Fig. 5). For targeted attacks, adversarial retraining significantly reduced the vulnerability to UAPs, but did not completely avoid it (particularly for the skin lesions image dataset, Fig. 6). Additionally, adversarial retraining requires high computational costs, as it is an iterative fine-tuning method. Simpler alternative methods, such as dimensionality reduction (e.g., principle component analysis), distributional detection (e.g., maximum mean discrepancy), and normalization detection (e.g., dropout randomization) are available; however, they are known to be easily detected as adversarial examples [15]. Despite the recent development in adversarial defenses, such as regularized surrogate loss optimization [27], the use of a discontinuous activation function [28], and improving the generalization of adversarial training with domain adaptation [29], many promising defense methods have failed [30]. Defending against adversarial attacks is a cat-and-mouse game [10]. Furthermore, properties inherent to image processing may cause misclassification. For instance, DNN-based image reconstructions are often performed to purify adversarial examples [31]; however, they cause image artifacts, resulting in misclassifications by DNNs [32]. It may be difficult to completely avoid security concerns caused by adversarial attacks.

The vulnerability to UAPs was confirmed in various model architectures. Vulnerability to UAPs may be a universal feature in DNNs. However, VGG16 and VGG19 were relatively robust against UAPs compared to the other model architectures. This result is consistent with the fact that shallower neural networks are more robust against adversarial attacks for the same task [33]. The use of these model architectures may be a simple solution for avoiding vulnerability to UAPs. However, such a solution may be unrealistic. The effect of the use of these model architectures on the decrease in $R_{f}$ and $R_{s}$ was limited (Tables 1 and 2). Simpler models may show a relatively low prediction performance. Given the tradeoffs between prediction performance and robustness against adversarial attacks [27], it may be difficult to develop DNNs with both high prediction performance and high robustness against UAPs.

Another simple solution for avoiding adversarial attacks is to render DNNs closed source and publicly unavailable; however, this hinders the accelerated development of medical DNNs and practical applications of DNNs to automated support for clinical diagnosis. Because the amount of medical image data is limited, collaboration among multiple institutions is required to achieve high diagnostic performance [34]. For similar reasons, medical DNNs are often developed by fine-tuning existing DNNs, such as VGG, ResNet, and Inception, pretrained using the ImageNet dataset (i.e., via transfer learning), although a previous study [34] debated the effect of transfer learning on the improvement in prediction performance for medical imaging; consequently, model architectures and model weights may be important. Furthermore, DNNs are aimed at real-world usage (e.g., automated support for clinical diagnosis). The assumption that DNNs are a closed source and publicly unavailable may be unrealistic. Even if DNNs are black-box (e.g., model architectures and weights are unknown and loss gradient is not accessible), adversarial attacks on DNNs may be possible. Several methods for adversarial attacks on black-box DNNs, which estimate adversarial perturbations using only model outputs (e.g., confidence scores), have been proposed [35–37]. The development and operation of secure, privacy-preserving, and federated DNNs are required in medical imaging [6].

## Conclusion

Our study is the first to show the vulnerability of DNN-based medical image classification to both nontargeted and targeted UAPs. Our findings emphasize that careful consideration is required in the development of DNNs for medical imaging and their practical applications. Inspired by the high prediction performance of DNNs, many studies have applied DNNs to medical image classification; however, they have ignored the vulnerability of UAPs. Our study highlights such facile applications of DNNs. Our findings enhance our understanding of the vulnerabilities of DNNs to adversarial attacks and may help increase the security of DNNs. UAPs are useful for reliability evaluation and for designing the operation strategy of medical DNNs.

## Supplementary Information

**Additional file 1:** Supplementary tables and figures.

## Data Availability

All data generated and analyzed during this study are included in this published article and its supplementary information files. The code and data used in this study are available from our GitHub repository: github.com/hkthirano/MedicalAI-UAP.

## Abbreviations

AKIEC: Actinic keratosis/Bowens disease (intraepithelial carcinoma)
BCC: Basal cell carcinoma
BKL: Benign keratosis (solar lentigo/seborrheic keratosis/lichen planus-like keratosis)
CNV: Neovascular membrane and associated subretinal fluid
DF: Dermatofibroma
DME: Diabetic macular edema with retinal-thickening-associated intraretinal fluid
DNN: Deep neural network
DRUSEN: Multiple drusen present in early age-related macular degeneration
DensNet: Dense convolutional network
FC: Fully connected
FGSM: Fast gradient sign method
ISIC: International Skin Imaging Collaboration
MEL: Melanoma
NM: Normal retina with preserved foveal contour and absence of retinal fluid/edema
NV: Melanocytic nevus
OCT: Optical coherence tomography
ResNet: Residual network
UAP: Universal adversarial perturbation
VASC: Vascular lesion
VGG: Visual geometry group
