# Supplementary material for: Universal adversarial attacks on deep neural networks for medical image classification
Source: BMC Med Imaging. 2021 Jan 7;21:9. doi: 10.1186/s12880-020-00530-y (PMC7792111; doi:10.1186/s12880-020-00530-y)
Supplement: Supplementary file 1 — Additional file 1: Supplementary tables and figures. [file 12880_2020_530_MOESM1_ESM.pdf]

Supplementary material of "Universal adversarial attacks on deep neural networks for medical image classification"

Hokuto Hirano[1], Akinori Minagi[1], Kazuhiro Takemoto[1*]
1) Department of Bioscience and Bioinformatics, Kyushu Institute of Technology, Iizuka, Fukuoka 820-8502, Japan
**Corresponding author's e-mail: takemoto@bio.kyutech.ac.jp*

Table S1: Training and test accuracies (%) of various DNN models for skin lesion, OCT, and chest X-ray image datasets.

Model architecture	Skin lesion		OCT		Chest X-ray	
	Training	Test	Training	Test	Training	Test
Inception V3	100.0%	87.7%	99.8%	95.5%	99.7%	97.6%
VGG16	99.3%	86.3%	99.2%	96.1%	99.2%	98.3%
VGG19	99.3%	85.7%	99.8%	95.8%	99.7%	98.3%
ResNet50	99.7%	88.2%	99.8%	95.9%	99.5%	99.1%
Inception ResNet V2	99.9%	86.3%	99.8%	94.9%	99.8%	97.4%
DenseNet 121	99.7%	88.5%	99.3%	96.0%	99.8%	99.1%
DenseNet 169	99.9%	88.7%	99.8%	96.3%	99.8%	98.7%

Table S2: Targeted attack success rates R_s (%) of targeted UAPs with $p = \infty$ against various DNN models to each target class.

Model architecture / Target class	Skin lesion		OCT		Chest X-ray	
	NV	MEL	NM	CNV	NORMAL	PNEUMONIA
Inception V3	88.4 (65.3)	73.9 (12.0)	97.5 (27.2)	99.3 (25.8)	93.5 (52.6)	90.7 (47.4)
VGG16	86.8 (71.4)	43.4 (8.4)	97.4 (26.1)	99.8 (25.2)	98.5 (50.4)	98.1 (49.6)
VGG19	89.7 (71.9)	65.2 (8.8)	98.9 (26.6)	99.6 (25.6)	99.1 (51.9)	98.7 (48.1)
ResNet50	87.1 (66.0)	51.3 (11.5)	98.7 (28.4)	99.1 (25.7)	96.7 (54.4)	93.5 (45.6)
Inception ResNet V2	80.8 (61.8)	58.2 (15.8)	97.8 (26.8)	96.2 (25.3)	98.0 (53.5)	89.4 (46.5)
DenseNet 121	88.4 (65.1)	69.8 (13.3)	97.8 (26.3)	98.5 (24.7)	95.2 (52.2)	86.5 (47.8)
DenseNet 169	87.9 (65.6)	62.1 (12.2)	97.4 (27.2)	99.1 (25.2)	95.0 (52.2)	91.7 (47.8)

R_s are for test images. $\zeta = 2\%$ for skin lesion and chest X-ray image datasets. $\zeta = 6\%$ for OCT image dataset. Values in brackets are R_s of random UAPs (random controls).

Table S3: Transferability of nontargeted UAPs with $p = 2$ and $\zeta = 6\%$ for OCT image dataset.

Source / Target	Inception V3	VGG16	VGG19	ResNet50	Inception ResNet V2	DenseNet 121	DenseNet 201
Inception V3	70.2%	0.6%	0.6%	1.8%	3.9%	1.4%	1.5%
VGG16	1.6%	72.4%	41.4%	2.6%	3.0%	1.6%	2.6%
VGG19	2.3%	49.4%	72.8%	2.9%	3.1%	1.9%	2.7%
ResNet50	1.6%	0.3%	0.7%	71.2%	2.3%	3.1%	2.8%
Inception ResNet V2	1.9%	0.4%	0.6%	1.5%	69.6%	1.2%	1.7%
DenseNet 121	1.2%	0.4%	0.6%	10.6%	6.3%	68.8%	12.2%
DenseNet 169	1.8%	0.5%	0.5%	2.0%	3.0%	5.1%	50.3%

Rows and columns indicate model architecture for which UAP was computed and architecture for which R_f was reported, respectively. R_f are for test images.

Table S4: Transferability of nontargeted UAPs with $p = 2$ and $\zeta = 4\%$ for the chest X-ray image dataset.

Source / Target	Inception V3	VGG16	VGG19	ResNet50	Inception ResNet V2	DenseNet 121	DenseNet 201
Inception V3	81.7%	2.6%	9.8%	3.5%	5.7%	2.8%	2.8%
VGG16	21.7%	49.8%	47.6%	24.1%	6.9%	17.2%	18.9%
VGG19	11.3%	43.9%	49.3%	11.3%	25.4%	6.9%	5.2%
ResNet50	7.4%	3.0%	4.4%	72.6%	7.4%	4.3%	1.9%
Inception ResNet V2	2.2%	3.0%	8.7%	2.8%	78.0%	4.4%	3.5%
DenseNet 121	6.7%	3.1%	2.8%	8.0%	9.1%	69.8%	2.0%
DenseNet 169	8.1%	3.9%	3.3%	5.7%	10.4%	5.0%	67.6%

Rows and columns indicate model architecture for which UAP was computed and architecture for which R_f was reported, respectively. R_f are for test images.

Table S5: Transferability of nontargeted UAPs with $p = 2$ and $\zeta = 4\%$ for the skin lesion image dataset.

Source / Target	Inception V3	VGG16	VGG19	ResNet50	Inception ResNet V2	DenseNet 121	DenseNet 201
Inception V3	92.2%	9.4%	9.7%	16.1%	38.1%	18.7%	18.8%
VGG16	36.2%	87.6%	72.8%	20.6%	29.3%	37.8%	29.1%
VGG19	43.6%	69.7%	89.2%	18.5%	22.6%	21.5%	27.9%
ResNet50	37.0%	9.2%	9.3%	91.9%	32.8%	25.7%	34.8%
Inception ResNet V2	35.3%	9.1%	9.5%	16.2%	94.5%	16.5%	17.4%
DenseNet 121	49.0%	9.4%	9.9%	20.1%	43.9%	93.8%	43.5%
DenseNet 169	57.8%	10.2%	10.2%	20.9%	49.0%	57.1%	93.8%

Rows and columns indicate model architecture for which UAP was computed and architecture for which R_f was reported, respectively. R_f are for test images.

Table S6: Transferability of targeted UAPs to MEL with $p = 2$ and $\zeta = 4\%$ for the skin lesion image dataset.

Source / Target	Inception V3	VGG16	VGG19	ResNet50	Inception ResNet V2	DenseNet 121	DenseNet 201
Inception V3	94.4%	8.5%	8.4%	12.0%	16.0%	13.8%	13.4%
VGG16	8.0%	40.4%	10.5%	10.7%	11.3%	11.8%	11.0%
VGG19	9.5%	10.0%	64.6%	10.7%	12.8%	12.7%	11.5%
ResNet50	13.4%	8.5%	8.6%	92.4%	16.2%	15.2%	13.4%
Inception ResNet V2	13.5%	8.7%	8.2%	12.1%	97.3%	13.9%	11.6%
DenseNet 121	13.7%	8.5%	8.7%	12.0%	14.1%	90.5%	14.5%
DenseNet 169	12.8%	9.2%	8.6%	15.4%	12.5%	24.3%	92.9%

Rows and columns indicate model architecture for which UAP was computed and architecture for which R_f was reported, respectively. R_f are for test images.

Table S7: Transferability of targeted UAPs to CNV with $p = 2$ and $\zeta = 6\%$ for the OCT image dataset.

Source / Target	Inception V3	VGG16	VGG19	ResNet50	Inception ResNet V2	DenseNet 121	DenseNet 201
Inception V3	95.9%	25.1%	25.2%	25.8%	28.2%	25.1%	24.4%
VGG16	25.9%	97.7%	87.4%	26.6%	26.9%	26.5%	25.1%
VGG19	25.8%	77.4%	97.5%	27.7%	27.1%	26.7%	25.1%
ResNet50	27.7%	25.0%	25.0%	98.5%	27.2%	25.1%	24.5%
Inception ResNet V2	28.5%	25.0%	25.1%	26.0%	96.2%	25.3%	24.4%
DenseNet 121	25.4%	25.1%	24.9%	26.5%	25.5%	88.1%	24.3%
DenseNet 169	26.2%	25.0%	25.3%	29.3%	27.3%	25.7%	92.7%

Rows and columns indicate model architecture for which UAP was computed and architecture for which R_f was reported, respectively. R_f are for test images.

Table S8: Transferability of targeted UAPs to PNEUMONIA with $p = 2$ and $\zeta = 4\%$ for the chest X-ray image dataset.

Source / Target	Inception V3	VGG16	VGG19	ResNet50	Inception ResNet V2	DenseNet 121	DenseNet 201
Inception V3	93.3%	50.2%	48.9%	50.2%	53.0%	49.8%	49.4%
VGG16	52.6%	95.0%	77.0%	54.3%	50.6%	54.8%	55.9%
VGG19	58.9%	90.0%	95.2%	59.1%	55.6%	56.3%	60.9%
ResNet50	52.0%	52.6%	51.7%	95.2%	51.9%	52.0%	51.1%
Inception ResNet V2	51.1%	49.6%	48.3%	49.1%	93.9%	49.3%	49.3%
DenseNet 121	53.3%	52.4%	51.5%	52.0%	53.7%	92.0%	51.1%
DenseNet 169	51.3%	52.0%	50.9%	52.4%	51.7%	52.6%	93.1%

Rows and columns indicate model architecture for which UAP was computed and architecture for which R_f was reported, respectively. R_f are for test images.

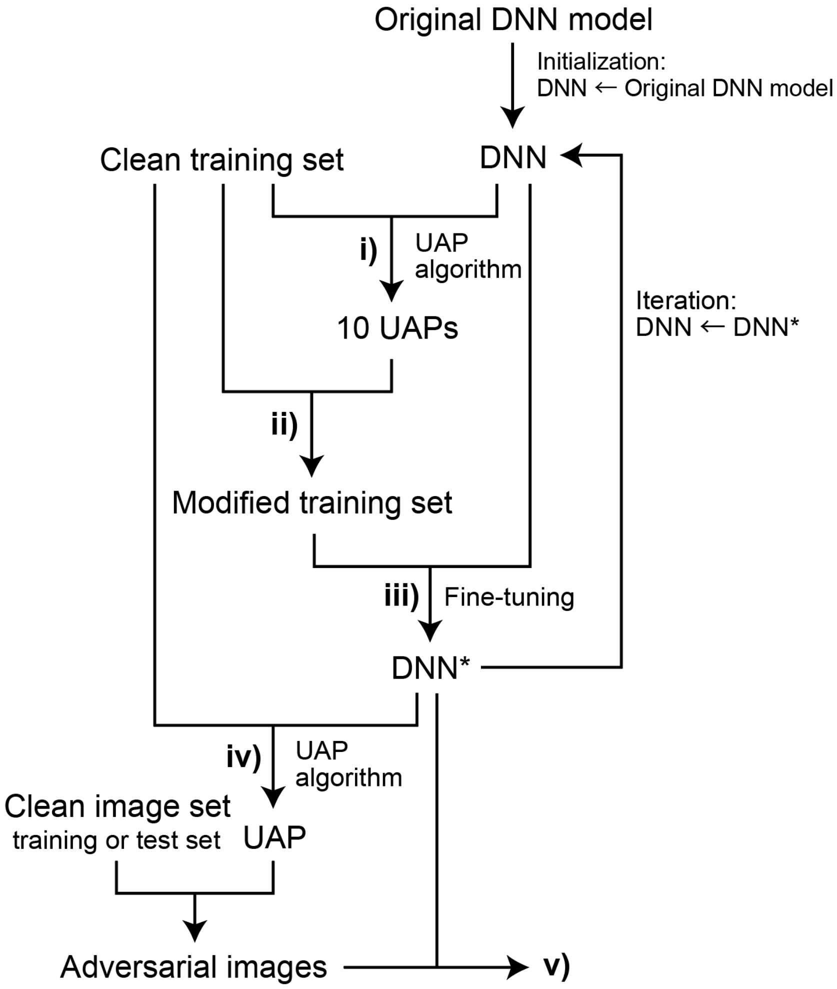

Fig. S1: Schematic diagram of adversarial retraining procedure.

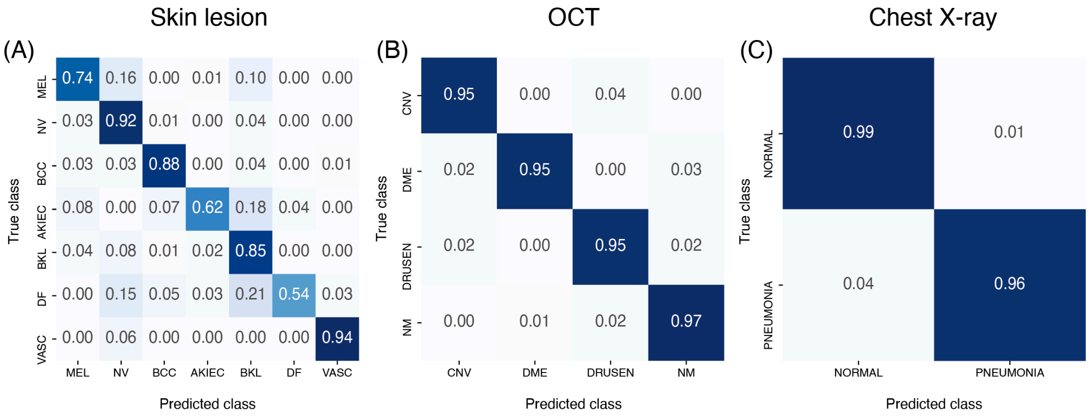

Fig. S2: Normalized confusion matrices for the Inception V3 models on the test images of skin lesion (A), OCT (B), and chest X-ray (C) image datasets.

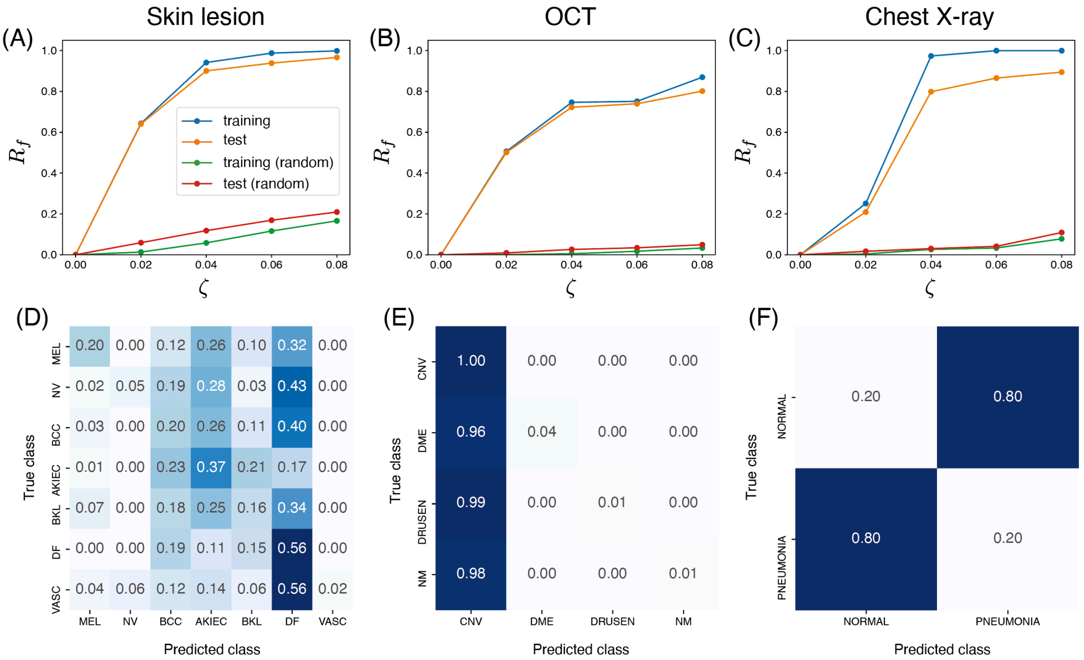

Fig. S3: Vulnerability to nontargeted UAPs with $p = \infty$. Line plots of the fooling rate R_f against Inception V3 model versus perturbation magnitude ζ for skin lesion (A), OCT (B), and chest X-ray (C) image datasets. Legend label indicates image set used for computing R_f. Additional argument "(random)" indicates that random UAPs were used instead of UAPs. Normalized confusion matrices for Inception V3 models attacked using UAPs on test images of skin lesion (D), OCT (E), and chest X-ray (F) image datasets are also shown. $\zeta = 4\%$ in (D) and (F). $\zeta = 6\%$ in (E).

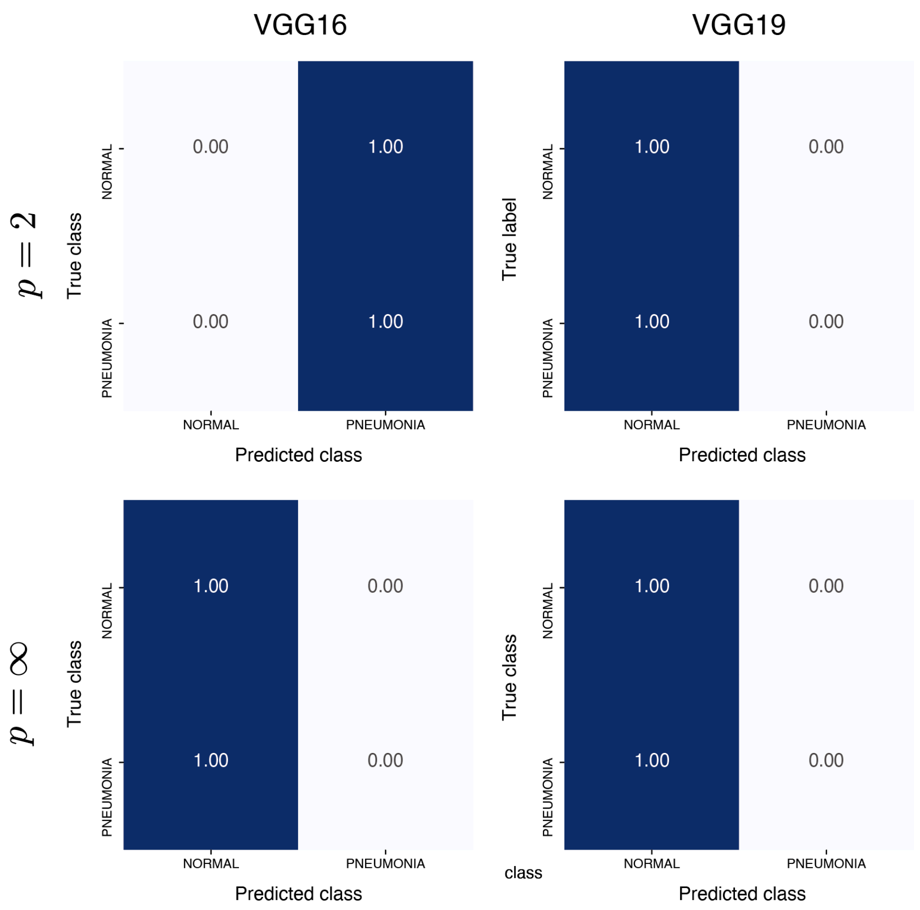

Fig. S4: Normalized confusion matrices for VGG16 (left panels) and VGG19 (right panels) models attacked using UAPs with $p = 2$ (top panels) and with $p = \infty$ (bottom panels) on test images of chest X-ray image dataset. $\zeta = 4\%$.

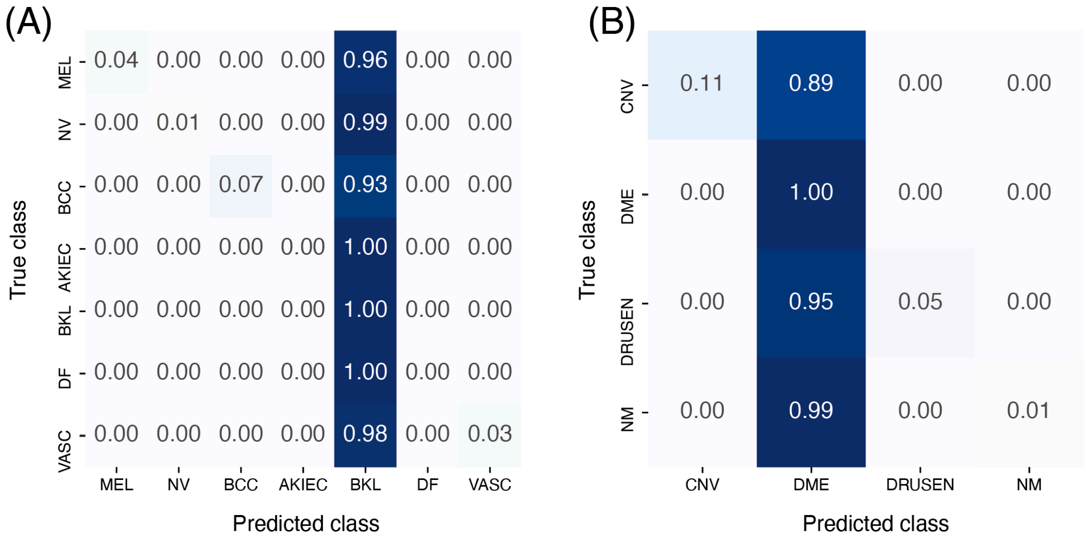

Fig. S5: Normalized confusion matrices for VGG16 (A) and ResNet50 (B) models attacked using UAPs with $p = 2$ on test images. Test images in (A) and (B) are from skin lesion and chest X-ray image datasets, respectively. $\zeta = 4\%$ in (A). $\zeta = 6\%$ in (B).

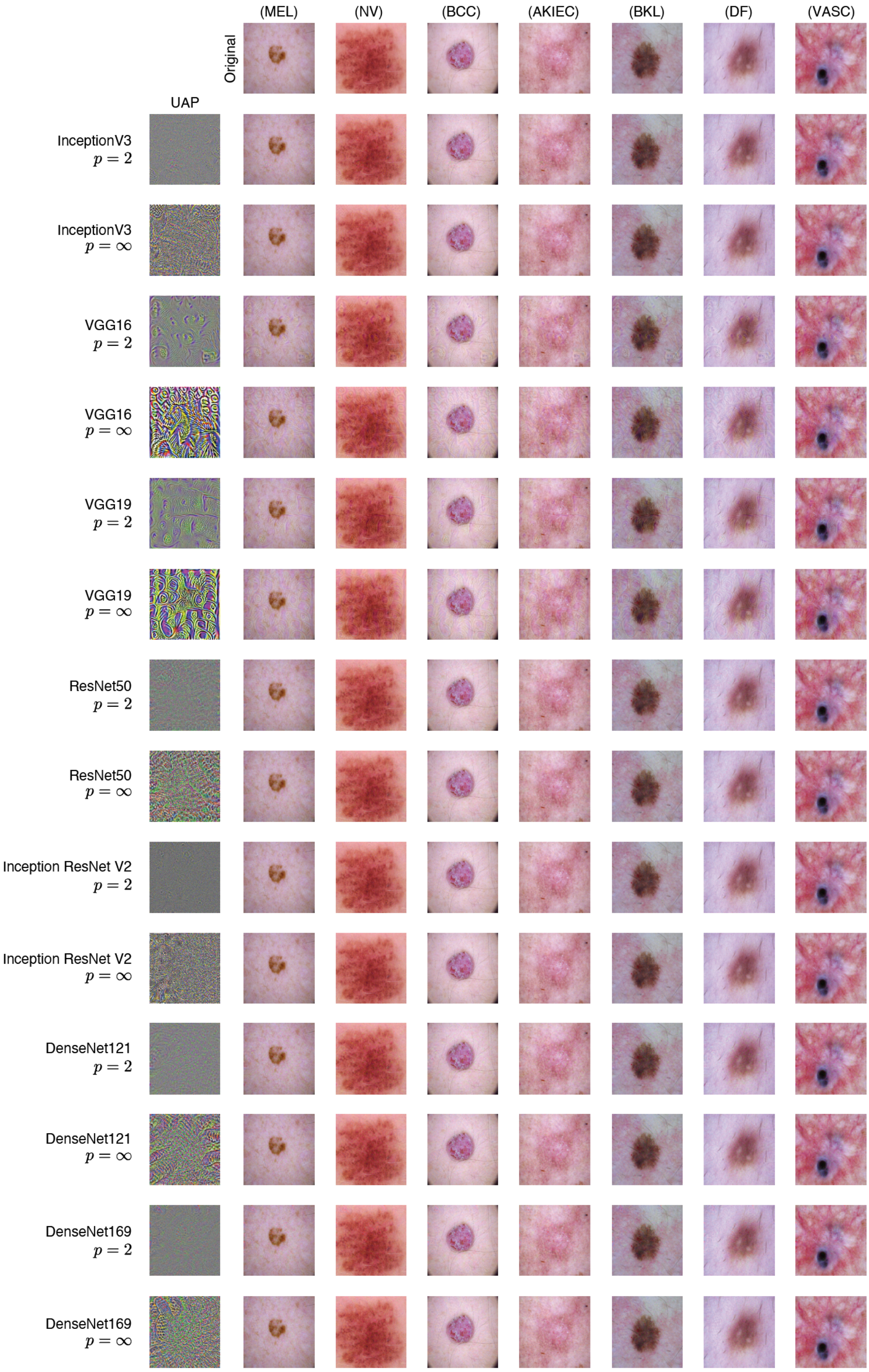

Fig. S6: Nontargeted UAPs with $p = 2$ and $p = \infty$ against various DNN models and their adversarial images for skin lesion image dataset. $\zeta = 4\%$. See Figure 2 for description of figure elements.

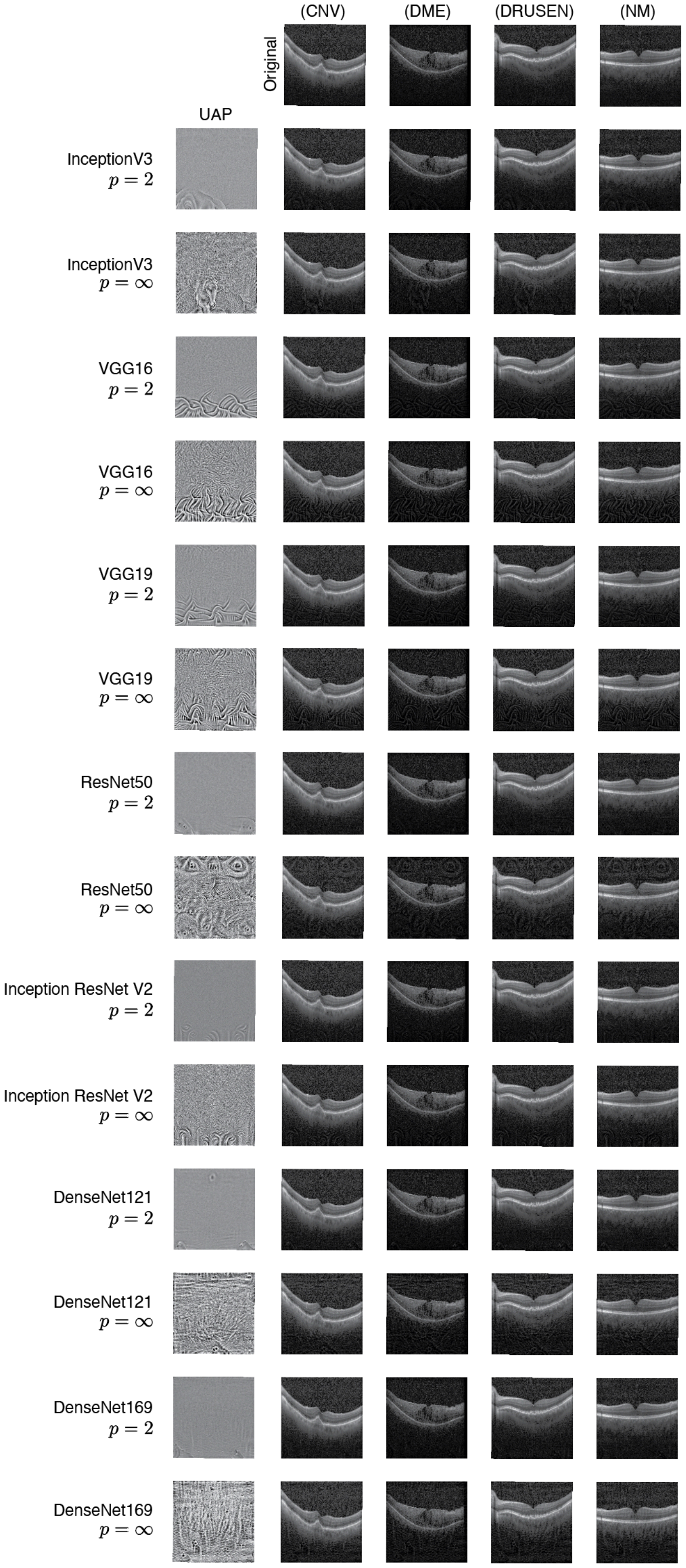

Fig. S7: Nontargeted UAPs with $p = 2$ and $p = \infty$ against various DNN models and their adversarial images for OCT image dataset. $\zeta = 6\%$. See Figure 2 for description of figure elements.

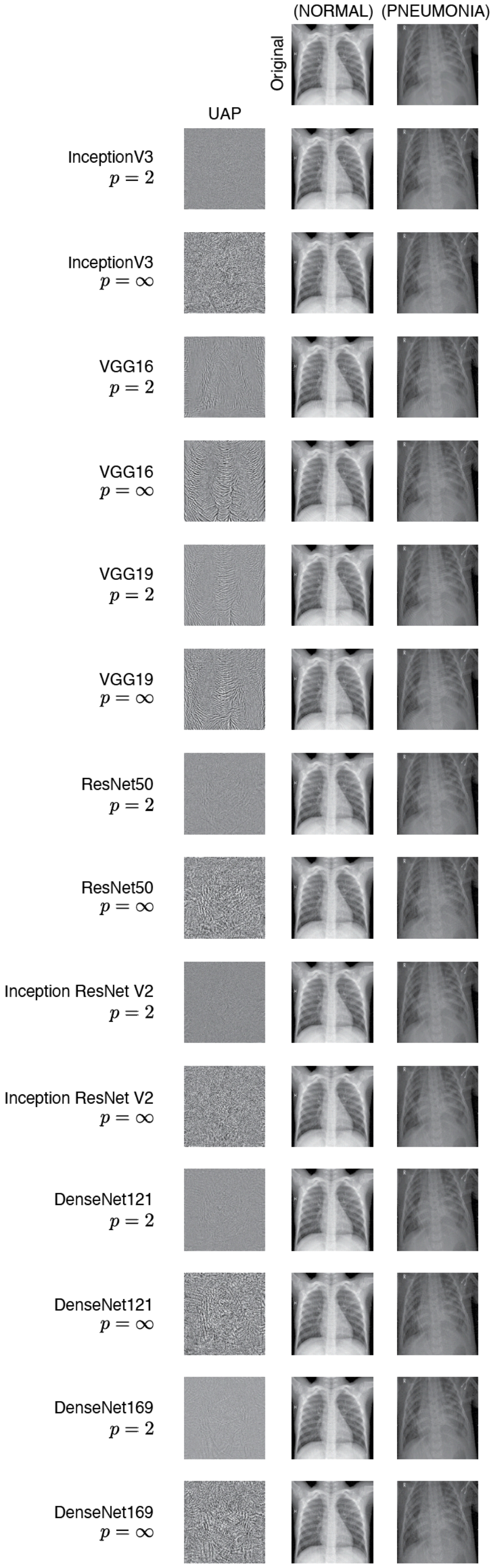

Fig. S8: Nontargeted UAPs with $p = 2$ and $p = \infty$ against various DNN models and their adversarial images for chest X-ray image dataset. $\zeta = 4\%$. See Figure 2 for description of figure elements.

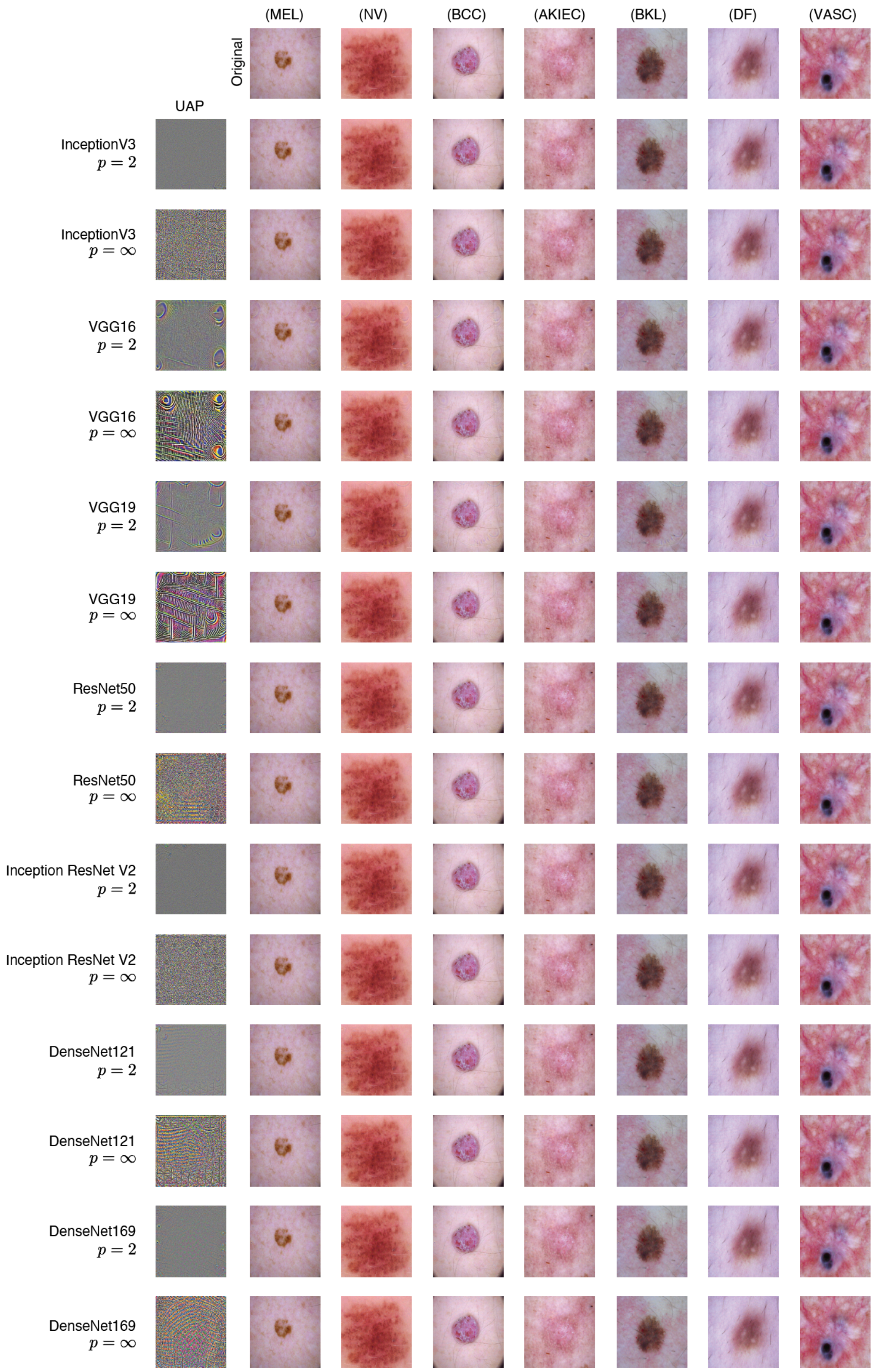

Fig. S9: Targeted UAPs to NV with $p = 2$ and $p = \infty$ against various DNN models and their adversarial images for skin lesion image dataset. $\zeta = 2\%$. See Figure 4 for description of figure elements.

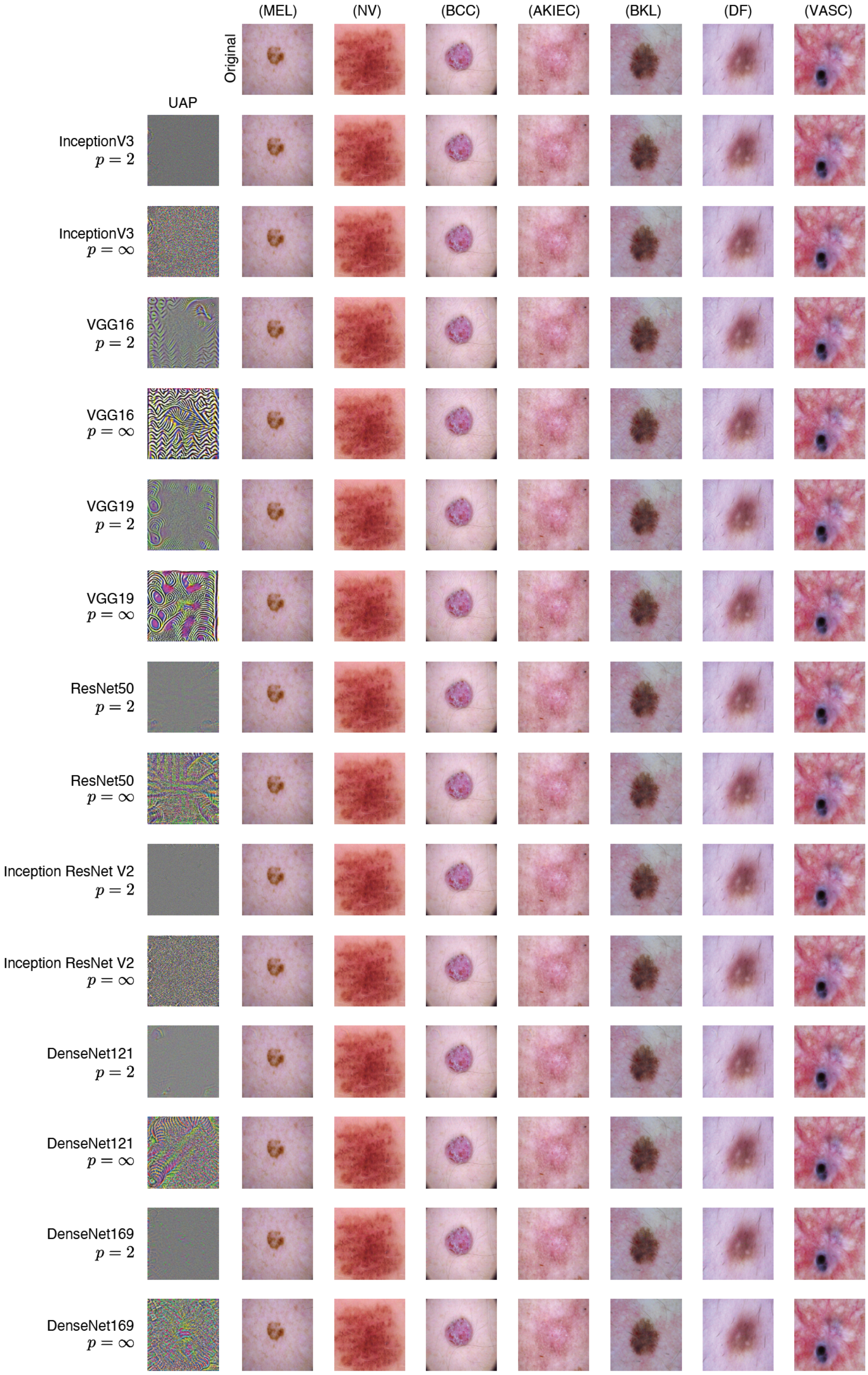

Fig. S10: Targeted UAPs to MEL with $p = 2$ and $p = \infty$ against various DNN models and their adversarial images for skin lesion image dataset. $\zeta = 2\%$. See Figure 4 for description of figure elements.

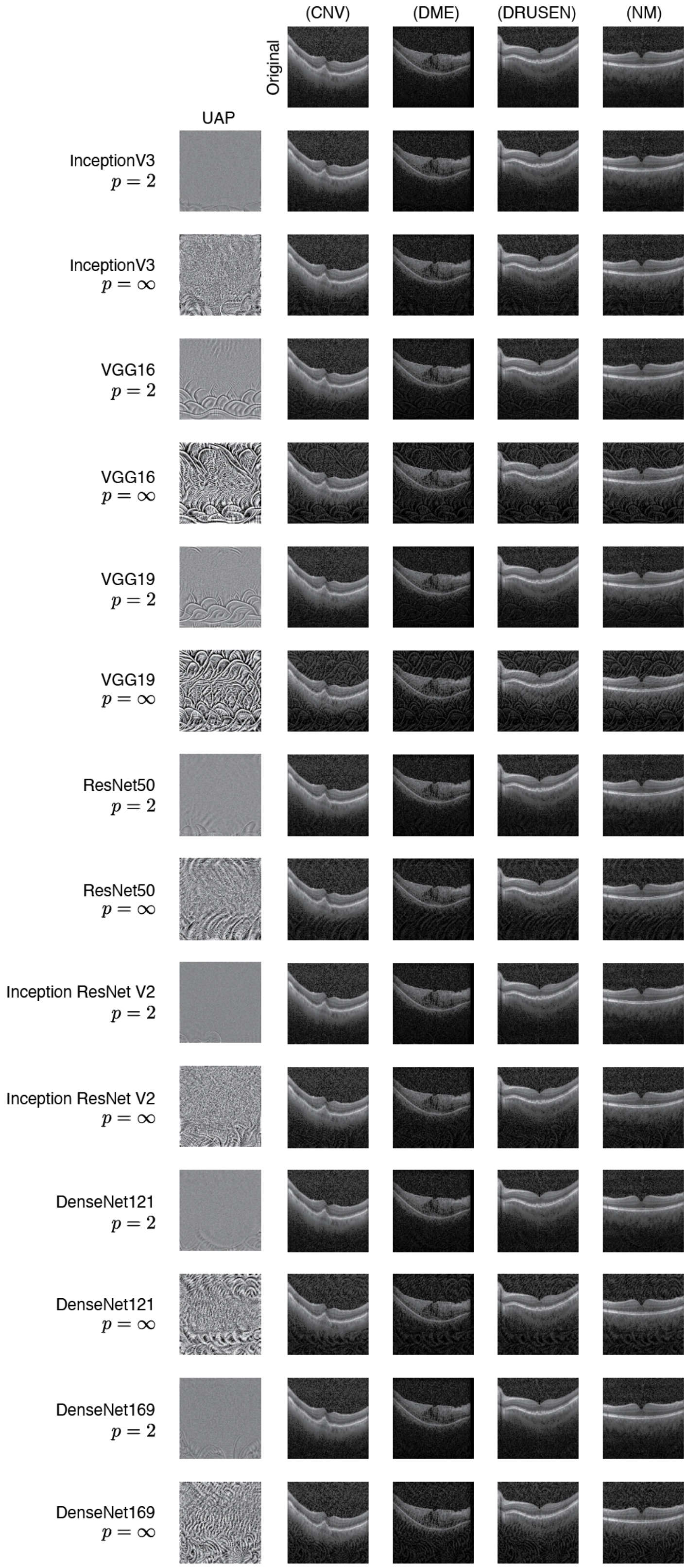

Fig. S11: Targeted UAPs to NM with $p = 2$ and $p = \infty$ against the various DNN models and their adversarial images for OCT image dataset. $\zeta = 6\%$. See Figure 4 for description of figure elements.

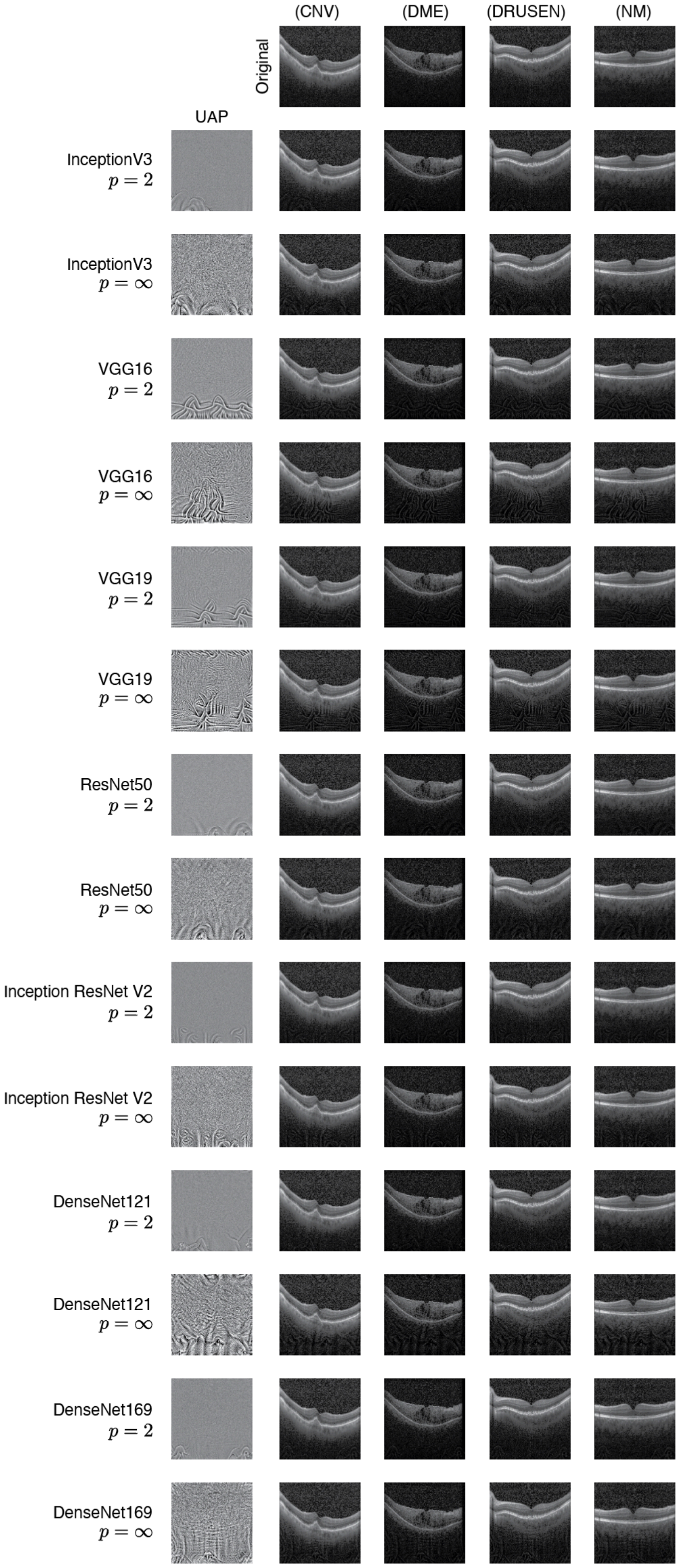

Fig. S12: Targeted UAPs to CNV with $p = 2$ and $p = \infty$ against the various DNN models and their adversarial images for OCT image dataset. $\zeta = 6\%$. See Figure 4 for description of figure elements.

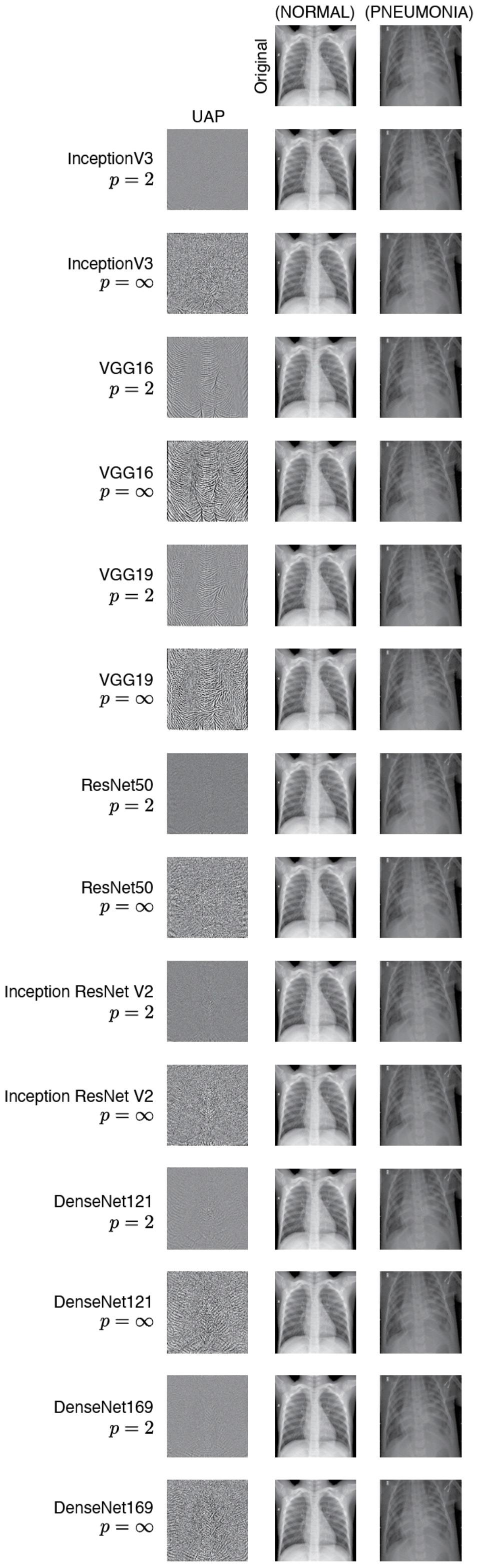

Fig. S13: Targeted UAPs to NORMAL with $p = 2$ and $p = \infty$ against the various DNN models and their adversarial images for chest X-ray image dataset. $\zeta = 2\%$. See Figure 4 for description of figure elements.

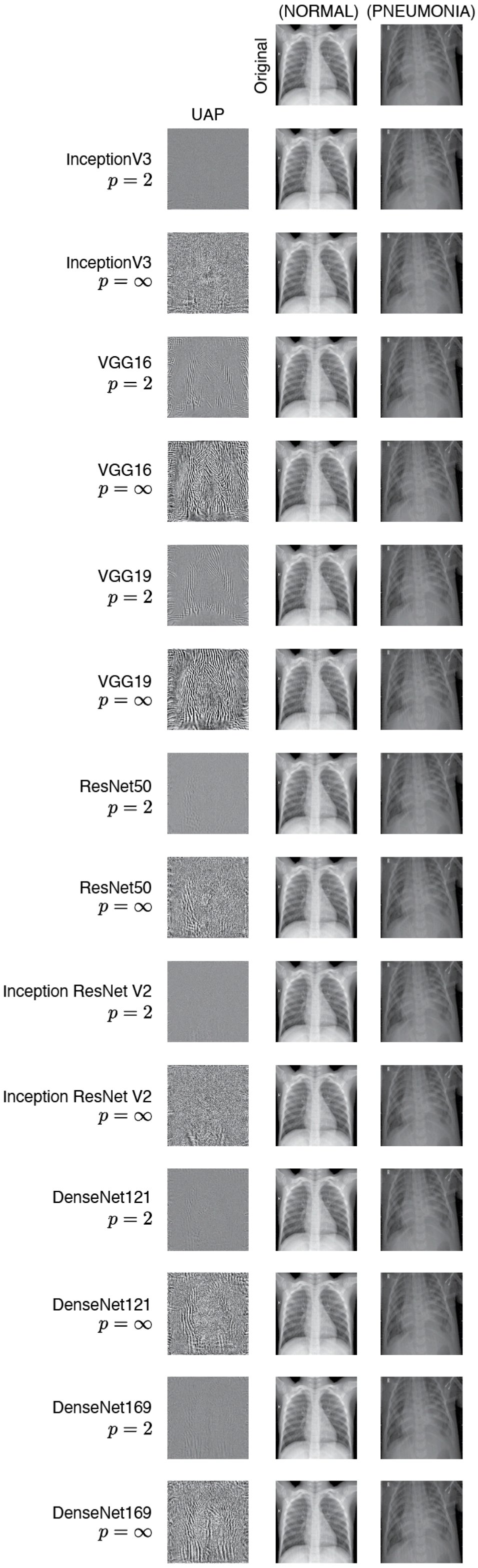

Fig. S14: Targeted UAPs to PNEUMONIA with $p = 2$ and $p = \infty$ against the various DNN models and their adversarial images for chest X-ray image dataset. $\zeta = 2\%$. See Figure 4 for description of figure elements.

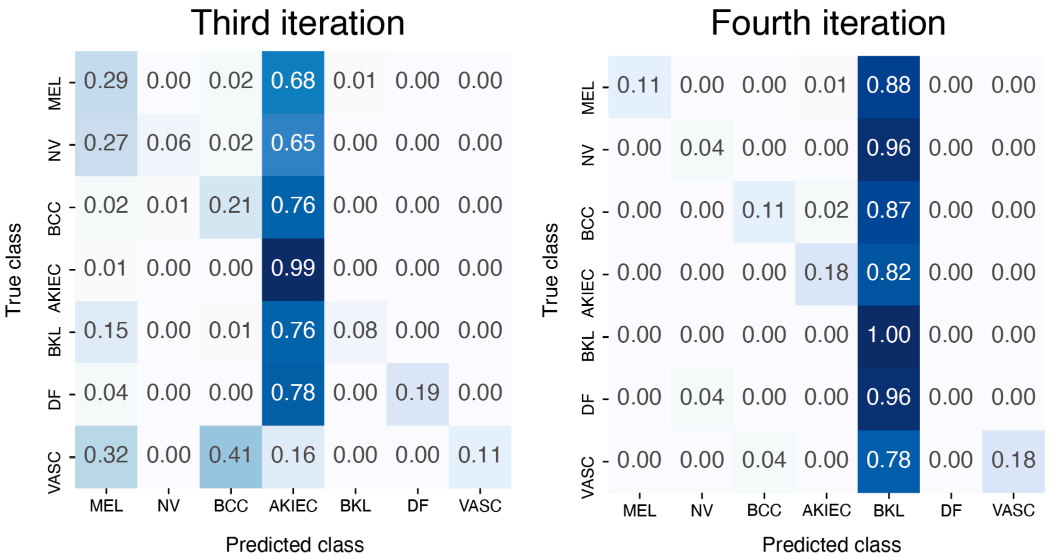

Fig. S15: Normalized confusion matrices for Inception V3 model attacked using nontargeted UAPs with $p = 2$ and $\zeta = 4\%$ on test images of skin lesion image dataset at third (left) and fourth (right) iterations of adversarial retraining.
